# Supplementary material for: Genetic evidence for a periplasmic protein as a third component for a subset of NtrYX family two-component systems
Source: J Bacteriol. 2026 Feb 13;208(3):e00521-25. doi: 10.1128/jb.00521-25 (PMC13001219; doi:10.1128/jb.00521-25)
Supplement: Supplemental figure legends — Legends for Figures S1 to S4. [file jb.00521-25-s0001.docx]

**Legends for Supplemental Figures**

**Supplemental Figure 1**. *rsmB* homologs are widely distributed, but some homologs

are found adjacent to a subset of *ntrY* homologs in β- and Ɣ-proteobacteria. A)

Distribution of *rsmB* within proteobacteria. Red lines represent orders that contain

genomes with genes encoding *rsmB*. Gray lines represent orders that do not. (B) Gene

presence/absence plot of *plrS* homologs vs *rsmB* homologs. Score ratios closer to 1

indicate a greater degree of homology to the queried genes. A score ratio below 0 indicates no homolog was detected in that genome. Solid green dots indicate the *plrS* and *rsmB* homologs are found within 5kb of each other in the genome. Orange dashed line indicates the arbitrary threshold for good homology (30% of the maximum bit score).

**Supplemental Figure 2**. Altering the atmospheric environment does not affect

transcription of the *plrSR* gene cluster. Graph of RNA transcripts measured using

RNAseq from *B. bronchiseptica* samples that were grown in ambient air conditions, 5% O2, 2% O2, and 5% CO2 (40). Data was visualized using the Integrative Genomic Viewer (49).

**Supplemental Figure 3**. Model for *plrPSR* mutants *in vitro*.

WT: Our data suggest that PlrR~P levels are low when *B. bronchiseptica* is grown in SS or on BG agar in the lab. Under these conditions, PlrP, by interacting with the PlrS PDC domain, inhibits PlrS phosphatase activity. PlrR may be phosphorylated by PlrS or another phosphodonor.

Δ*plrP*: *plrP* is essential *in vitro*. In its absence, PlrS phosphatase activity is high. The other phosphodonor cannot compete with the strong phosphatase activity of PlrS, and hence PlrR~P levels are insufficient for cell viability.

ΔPDC: Our data suggest that PlrS lacking the PDC domain lacks phosphatase

activity. In this strain, PlrR~P levels could be the same or higher than in the wild-type strain *in vitro*.

ΔPDC Δ*plrP*: *plrP* can be deleted in the ΔPDC strain because PlrS ΔPDC lacks

phosphatase activity, hence PlrP is not required to inhibit PlrS phosphatase

activity.

PlrSH521Q: PlrS cannot be phosphorylated with the H521Q substitution, and hence cannot phosphorylate PlrR. PlrR must be phosphorylated by another molecule in this strain, because at least a low level of PlrR~P is required for viability *in vitro*.

PlrSH521Q ΔplrP: The fact that *plrP* can be deleted in the PlrSH521Q mutant indicates that PlrSH521Q must also be defective for phosphatase activity.

PlrSN525A: PlrSN525A is defective for phosphatase activity. (This mutant is likely also

defective for kinase activity, as evident from the in vitro biochemical analyses and the fact that it is defective *in vivo*.)

PlrSN525A Δ*plrP*: *plrP* can be deleted in the PlrSN525A mutant, because PlrSN525A

lacks phosphatase activity.

Δ*plrS* PlrRD52E: This mutant is viable. PlrRD52E is able to complement

the Δ*plrS* mutation *in vitro*, but only incompletely *in vivo*. These data suggest that PlrRD52E is not as active as PlrR~P, and that high levels of PlrR~P are required *in vivo*.

Δ*plrS* PlrRD52E Δ*plrP*: This mutant is viable in vitro because PlrRD52E functions as if phosphorylated, at least as if phosphorylated at a low level. PlrRD52E function is independent of PlrS.

**Supplemental Figure 4**. Model for plrPSR mutants *in vivo*.

WT: Our data suggest that PlrR~P levels are high when *B. bronchiseptica* is growing/persisting in the LRT. Under these conditions, PlrP, by interacting with the PlrS PDC domain, likely inhibits PlrS phosphatase activity, although its necessity for this in the LRT is unknown. It is also possible that PlrS functions as a kinase in the LRT in a manner independent of the PDC domain (and PlrP).

Δ*plrP*: We do not know if *plrP* is essential *in vivo*, because we cannot construct a Δ*plrP*

mutant.

ΔPDC: If PlrS lacking the PDC domain lacks phosphatase activity, as the data suggest,

then the ΔPDC mutant should not be defective (and it is not) in the LRT where a high level of PlrR~P is required.

ΔPDC Δ*plrP*: *plrP* can be deleted in the ΔPDC strain because PlrS ΔPDC

lacks phosphatase activity, hence PlrP is not required to inhibit PlrS phosphatase

activity.

PlrSH521Q: PlrS cannot be phosphorylated with the H521Q substitution, and hence

cannot phosphorylate PlrR. This strain is severely defective in the LRT, where a high level of PlrR~P is required. The phosphodonor that phosphorylates PlrR *in vitro* is either not present, or not present at high enough levels, when the bacteria are in the LRT.

PlrSH521Q Δ*plrP*: This mutant is likely severely defective *in vivo*, but was not tested in this study.

PlrSN525A: PlrSN525A is defective for phosphatase activity, and also (somewhat) defective for kinase activity. It is defective for persistence in the LRT, but not as defective as the PlrSH521Q mutant, suggesting that it retains some kinase activity.

PlrSN525A Δ*plrP*: This mutant is NOT defective in the LRT, indicating that

PlrR~P levels must be high in this strain when the bacteria are in the LRT. This result suggests that, at least when PlrS contains the N525A substitution, PlrP inhibits PlrS kinase activity in the LRT. In the absence of PlrP, PlrSN525A kinase activity is high and hence PlrR~P levels are high.

Regardless of how this mutant is behaving mechanistically, the fact that the PlrSN525A mutant and the PlrSN525A Δ*plrP* mutants have different phenotypes in the LRT indicates that, at least in this mutant context, PlrP affects PlrS activity *in vivo*.

Δ*plrS* PlrRD52E: This mutant is modestly defective for persistence in the LRT, likely because PlrRD52E is not as active as PlrR~P and that high levels of PlrR~P are required in the LRT.

Δ*plrS* PlrRD52E Δ*plrP*: This mutant would likely be modestly defective for persistence in the LRT. It was not tested in this study.
