# Supplementary figures and images for: Genetic evidence for a periplasmic protein as a third component for a subset of NtrYX family two-component systems
Source: J Bacteriol. 2026 Feb 13;208(3):e00521-25. doi: 10.1128/jb.00521-25 (PMC13001219; doi:10.1128/jb.00521-25)

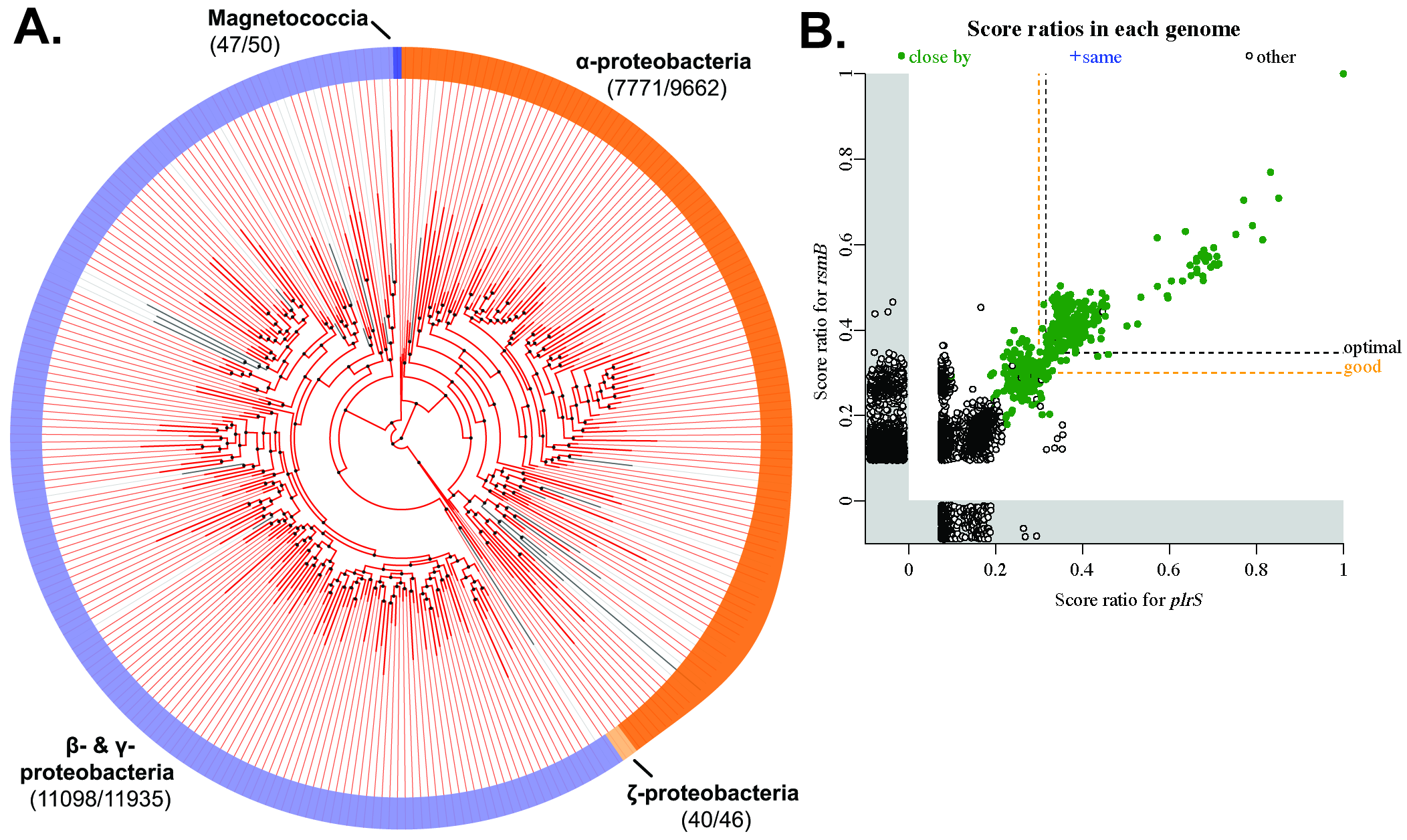

Supplement: Figure S1 — rsmB homologs are widely distributed, but some homologs are found adjacent to a subset of ntrY homologs in β- and γ-proteobacteria. [file jb.00521-25-s0002.tif]

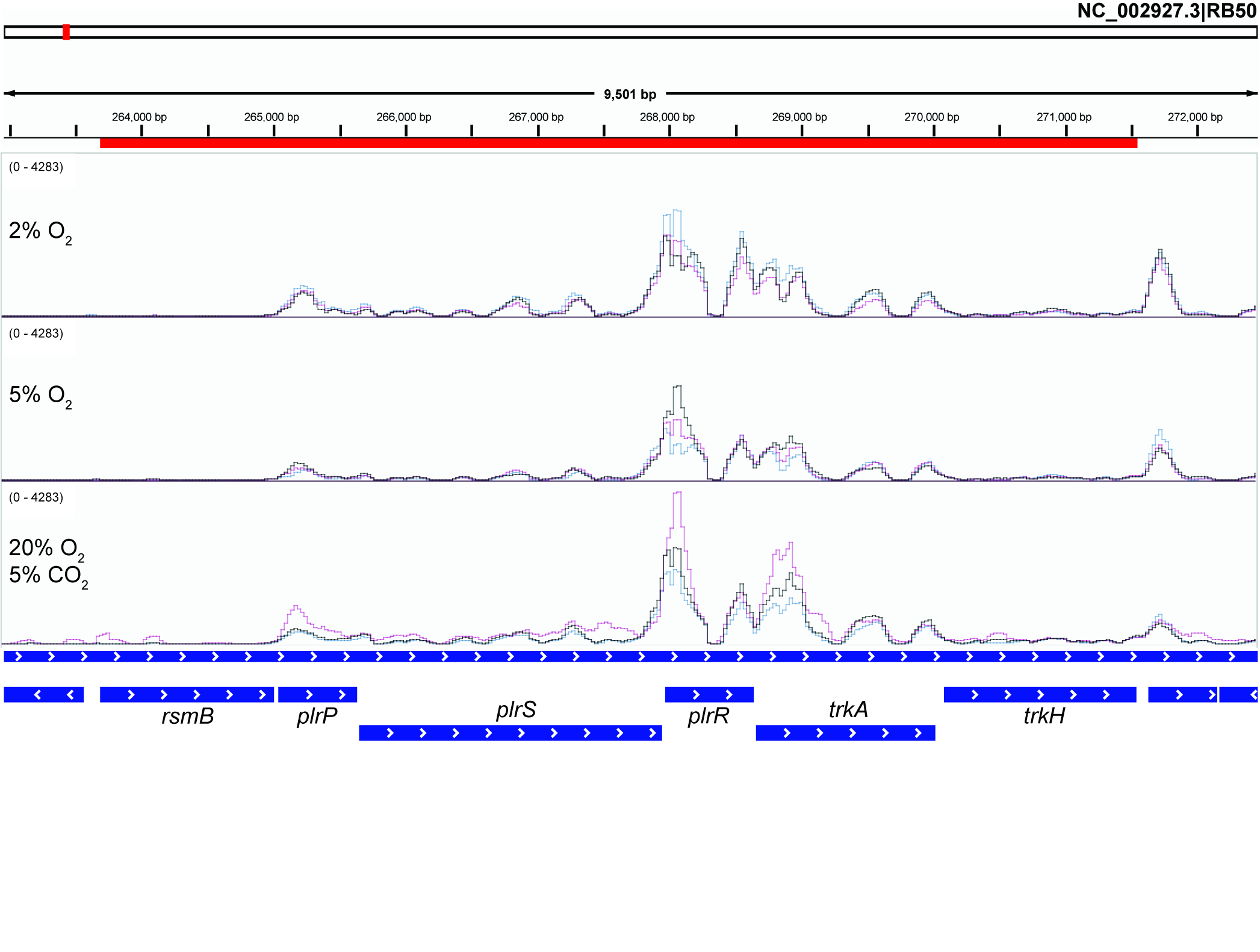

Supplement: Figure S2 — Altering the atmospheric environment does not affect transcription of the plrSR gene cluster. [file jb.00521-25-s0003.tif]

In SS medium, exp phase growth, 37 C

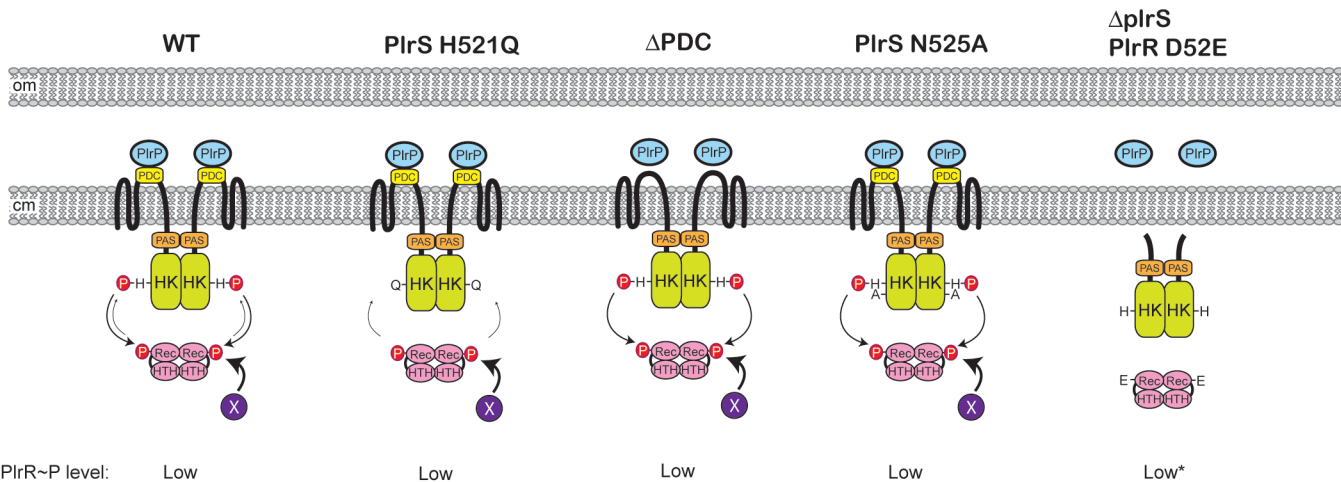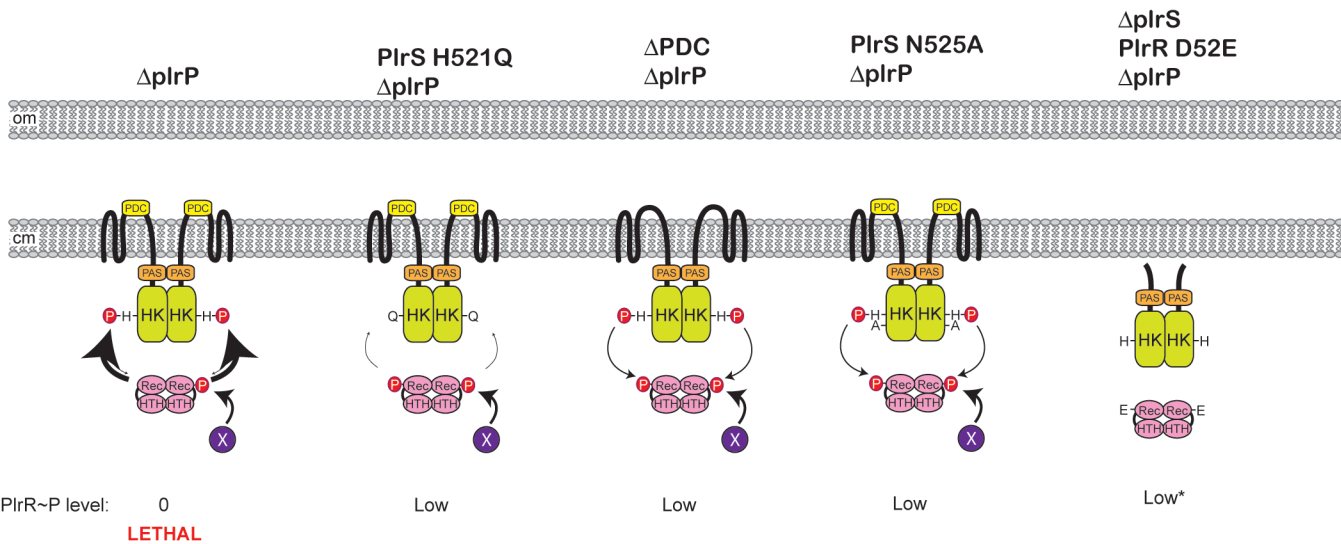

Supplement: Figure S3 — Model for plrPSR mutants in vitro. [file jb.00521-25-s0004.pdf]

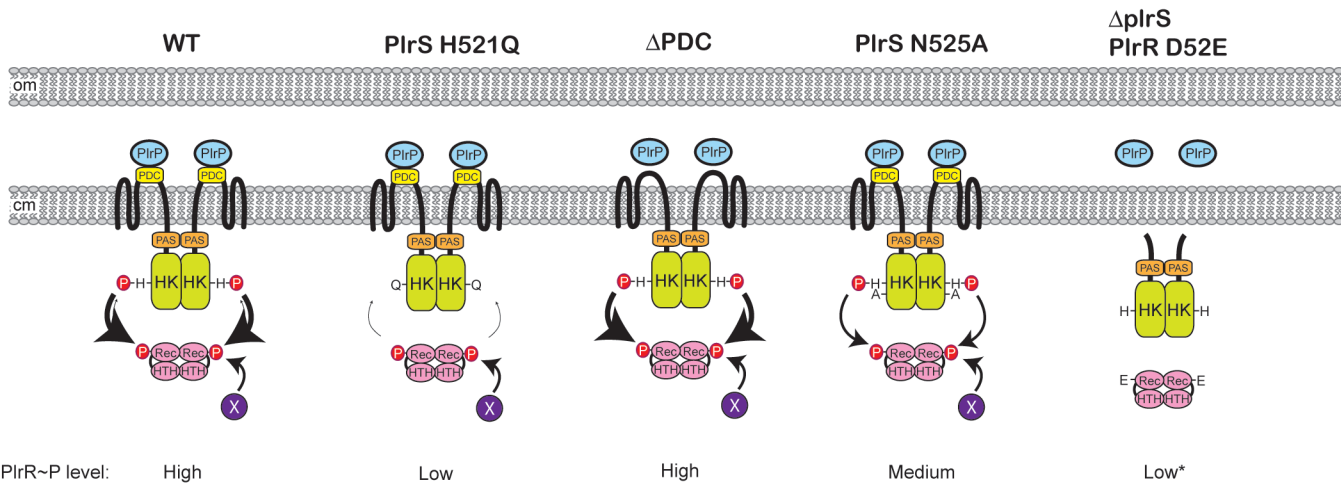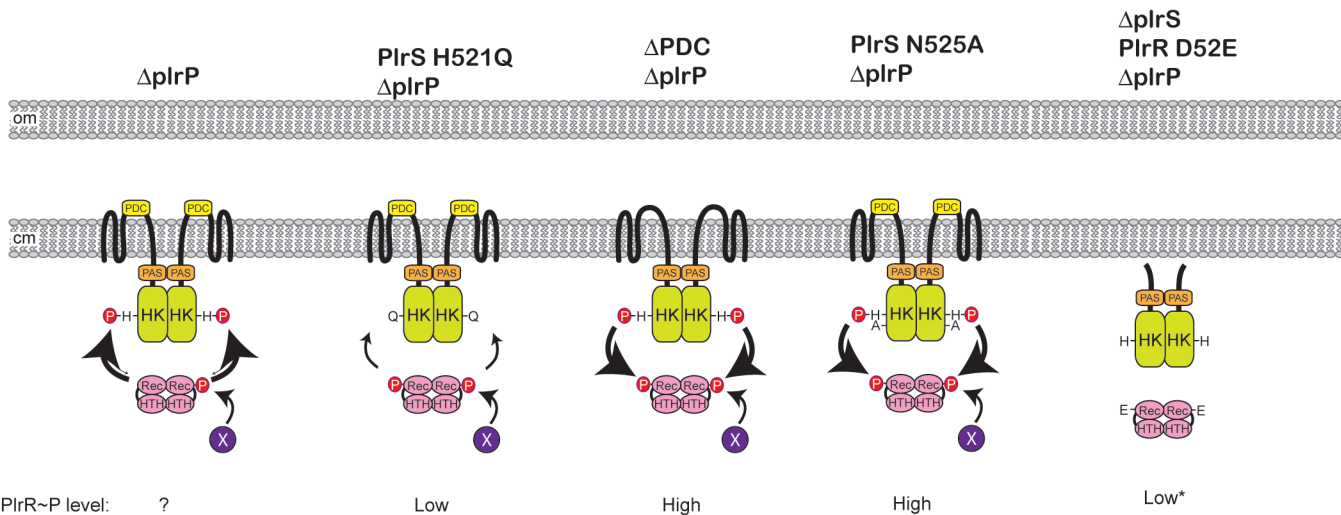

Supplement: Figure S4 — Model for plrPSR mutants in vivo. [file jb.00521-25-s0005.pdf]
