# Supplementary material for: Genetic evidence for a periplasmic protein as a third component for a subset of NtrYX family two-component systems
Source: J Bacteriol. 2026 Feb 13;208(3):e00521-25. doi: 10.1128/jb.00521-25 (PMC13001219; doi:10.1128/jb.00521-25)
Supplement: Table S1 — Strain and plasmid table. [file jb.00521-25-s0006.docx]

Supplemental Table 1. Strains and plasmids used in this study

| Bacterial Strains | Description | Reference |
| --- | --- | --- |
| *B. bronchiseptica* strains | | |
| RB50 (WT) | Wild-type *B. bronchiseptica* strain isolated from nares of a naturally infected rabbit; Sm^R^ | (1) |
| ∆*plrS* | RB50 containing an in-frame deletion in *plrS* of amino acids 5-198; Sm^R^ | (2) |
| *att*Tn*7*::P*_bvgA_*-*gfp* | RB50 containing pUC*gfp*-P*_bvgA_* at the *att*Tn7 site; Sm^R^ Km^R^ Sm^R^ Km^R^ | This study |
| ∆*plrS att*Tn*7*::P*_bvgA_*-*gfp* | ∆*plrS* containing pUC*gfp*-P*_bvgA_* at the *att*Tn7 site; Sm^R^ Km^R^ | This study |
| *att*Tn*7*::P*_rsmB_*-*gfp* | RB50 containing pUC*gfp*-P*_rsmB_* at the *att*Tn*7* site; Sm^R^ Km^R^ | This study |
| *att*Tn*7*::P*_plrP_*-*gfp* | RB50 containing pUC*gfp*-P*_plrP_* at the *att*Tn*7* site; Sm^R^ Km^R^ | This study |
| *att*Tn*7*::P*_plrR_*-*gfp* | RB50 containing pUC*gfp*-P*_plrR_* at the *att*Tn*7* site; Sm^R^ Km^R^ | This study |
| ∆*plrS att*Tn*7*::P*_rsmB_*-*gfp* | *∆plrS* containing pUC*gfp*-P*_rsmB_* at the *att*Tn*7* site; Sm^R^ Km^R^ | This study |
| ∆*plrS att*Tn*7*:: P*_plrP_*-*gfp* | *∆plrS* containing pUC*gfp*-P*_plrP_* at the *att*Tn*7* site; Sm^R^ Km^R^ | This study |
| ∆*plrS att*Tn*7*::P*_plrR_*-*gfp* | *∆plrS* containing pUC*gfp*-P*_plrR_* at the *att*Tn*7* site; Sm^R^ Km^R^ | This study |
| ∆*rsmB* | RB50 containing a deletion of 778bp within *rsmB* (nucleotides 8-1119); Sm^R^ | This study |
| ∆*trkAH* | RB50 containing a deletion of 2968bp within *trkAH* (nucleotide 13 of *trkA* through nucleotide 501 of *trkH*); Sm^R^ | This study |
| PlrS_H521Q_ | RB50 containing the H521Q point mutation in PlrS; Sm^R^ | (2) |
| PlrS_H521Q_ ∆*plrP* | PlrS_H521Q_ containing an in-frame deletion of 474bp within *plrP* (nucleotides 127-600); Sm^R^ | This study |
| ∆PDC | RB50 containing an in-frame deletion of 258bp encoding the PDC domain within *plrS* (nucleotides 463-720); Sm^R^ | This study |
| ∆PDC ∆*plrP* | ∆PDC containing an in-frame deletion of 474bp within *plrP* (nucleotides 127-600); Sm^R^ | This study |
| PlrR_D52E_ | RB50 containing an in-frame deletion of 678bp within *plrR* (nucleotides 4-684) and expression of *plrR* encoding the point mutation D52E at the *att*Tn*7* site; Sm^R^ Km^R^ | (3) |
| ∆*plrS* PlrR_D52E_ | ∆*plrS* containing an in-frame deletion of 678bp within *plrR* (nucleotides 4-684) and expression of *plrR* encoding the point mutation D52E at the *att*Tn*7* site; Sm^R^ Km^R^ | (3) |
| PlrR_D52E_ *∆plrP* | PlrR_D52E_ containing an in-frame deletion of 474bp within *plrP* (nucleotides 127-600); Sm^R^ | This study |
| PlrS_N525A_ | RB50 containing the N525A point mutation in PlrS; Sm^R^ | (4) |
| PlrS_N525A_ ∆*plrP* | PlrS_N525A_ containing an in-frame deletion of 474bp within *plrP* (nucleotides 127-600); Sm^R^ | This study |
| *E. coli* strains | | |
| DH5α | Molecular cloning strain | ThermoFisher |
| RHO3 | Conjugation strain (DAP auxotroph) | (5) |
| Plasmids | | |
| pEG7S | Suicide allelic exchange plasmid for *B. bronchiseptica* used to generate in-frame deletions; confers sucrose sensitivity through *sacB*; Gm^R^ Ap^R^ | (6) |
| pUC*gfp*MAB | *att*Tn*7*-site directed integration plasmid containing *gfp;* designed for assessing promoter activity; Km^R^ Ap^R^ | (7) |
| pTNS3 | *att*Tn*7* transposase expression vector containing *tnsABCD*; Ap^R^ | (5) |
| pUC*gfp*-P*_bvgA_* | pUC*gfp*MAB plasmid containing the 360bp upstream of *bvgA* integrated upstream of *gfp*; Km^R^ Ap^R^ | This study |
| pUC*gfp*-P*_rsmB_* | pUC*gfp*MAB plasmid containing the first 157bp of BB0261, the 107bp intergenic region between BB0261 and *rsmB,* and the first 54bp of *rsmB* (318bp total) integrated upstream of *gfp*; Km^R^ Ap^R^ | This study |
| pUC*gfp*-P*_plrP_* | pUC*gfp*MAB plasmid containing the final 250bp of *rsmB,* the 18bp intergenic region between *rsmB* and *plrP*, and the first 126bp of *plrP* (394bp total) integrated upstream of *gfp*; Km^R^ Ap^R^ | This study |
| pUC*gfp*-P*_plrR_* | pUC*gfp*MAB plasmid containing the final 238bp of *plrS* and the 10bp intergenic region between *plrS* and plrR (248bp total) integrated upstream of *gfp.* Km^R^ Ap^R^ | This study |
| pEG7S-∆*rsmB* | pEG7S-derivative plasmid containing ~500bp homology arms surrounding nucleotides 8-1119 of *rsmB;* Gm^R^ Ap^R^ | This study |
| pEG7S-∆*plrP* | pEG7S-derivative plasmid containing ~500bp homology arms surrounding nucleotides 126-600 of *plrP*; Gm^R^ Ap^R^. | This study |
| pEG7S-∆*trkAH* | pEG7S-derivative plasmid containing ~500bp homology arms surrounding nucleotide 13 of *trkA* through nucleotide 501 of *trkH;* Gm^R^ Ap^R^ | This study |
| pEG7S-∆PDC | pEG7S-derivative plasmid containing ~500bp homology arms surrounding nucleotide 463-720 of *plrS;* Gm^R^ Ap^R^ | This study |

**References**

1. Cotter PA, Miller JF. 1994. BvgAS-mediated signal transduction: analysis of phase-locked regulatory mutants of *Bordetella bronchiseptica* in a rabbit model. Infection and Immunity 62:3381–3390.

2. Kaut CS, Duncan MD, Kim JY, Maclaren JJ, Cochran KT, Julio SM. 2011. A Novel Sensor Kinase Is Required for Bordetella bronchiseptica To Colonize the Lower Respiratory Tract. Infect Immun 79:3216–3228.

3. Bone MA, Wilk AJ, Perault AI, Marlatt SA, Scheller EV, Anthouard R, Chen Q, Stibitz S, Cotter PA, Julio SM. 2017. Bordetella PlrSR regulatory system controls BvgAS activity and virulence in the lower respiratory tract. Proc Natl Acad Sci 114:E1519–E1527.

4. Barr SA, Kennedy EN, McKay LS, Johnson RM, Ohr RJ, Cotter PA, Bourret RB. 2023. Phosphorylation chemistry of the Bordetella PlrSR TCS and its contribution to bacterial persistence in the lower respiratory tract. Mol Microbiol 119:174–190.

5. López CM, Rholl DA, Trunck LA, Schweizer HP. 2009. Versatile dual-technology system for markerless allele replacement in *Burkholderia pseudomallei*. Appl Environ Microbiol 75:6496–6503.

6. Akerley BJ, Cotter PA, Miller JF. 1995. Ectopic expression of the flagellar regulon alters development of the *Bordetella*-host interaction. Cell 80:611–620.

7. Sobran MA, Cotter PA. 2019. The BvgS PAS Domain, an Independent Sensory Perception Module in the Bordetella bronchiseptica BvgAS Phosphorelay. J Bacteriol 201:10.1128/jb.00286-19.
